# Extracts from a Doctor’s Ledger

**Published:** 1908-03

**Authors:** B. H. Colegrove

**Affiliations:** Buffalo, N. Y.


### Extracts from a Doctor's Ledger

By B. H. COLEGROVE, Buffalo, N. Y.

THE following matter has recently been gathered from the back part of a ledger kept by the late Dr. Bela H. Colegrove of Sardinia, N. Y., in the years 1847 and 1848. Most of the material in this old ledger was written in the year 1868, when Dr. Colegrove was 71 years of age and after he had been engaged in the practice of medicine some fifty years. To many of the older members of the medical profession who knew Dr. Colegrove personally, as well as to those younger in the ranks, these extracts I trust will prove of interest. They hark back to the pioneer days when horse and saddle-bags were the vogue, and when the physician who essayed a country practice over the rough roads and bridle paths of that period had to be made of heroic fiber in order to succeed. Dr. Colegrove was successful in an eminent degree. As a surgeon he was rated as without a superior in his region, and there are those still living in Western New York who remember his operative work, either in their own behalf or that of friends, with thankful regard. Dr. Colegrove's home was in Sardinia, Erie County, thirty miles south of Buffalo. The following are some of the extracts:

According to the record preserved in my father's family Bible and copied into my own, I was born at Coventry, Rhode Island, April 2, 1797. About the year 1816, I commenced to learn the medical profession in the office of Dr. Thomas Hubbard, of Pomfret, Conn. Dr. Hubbard, at that time, was at the head of the profession in all that region of country, especially as a surgeon. He was one of the noblest specimens of manhood, physically as well as mentally, that I remember ever to have seen. In the later period of his life he received the appointment of professor of surgery in Yale College, at New Haven, where he died somewhere about 1840.

I had as a fellow student in his office, George McClellan, of Thompson, Conn., father of General George B. McClellan, and with him was a member of the medical class in the University of Pennsylvania, at Philadelphia, in the session of 1818-19. I received the degree of doctor of medicine the spring following, from the College of Physicians and Surgeons, in the city of New York. Beside McClellan and myself as students in the office of Dr. Hubbard, at Pomfret, were William Webb, of Windham, Conn., William P. Eaton, of Plainfield, Josiah Rose, of Coventry, Conn., and Francis Colwell, of Smithfield, R. I. Three of these went with McClellan and myself to Philadelphia to lectures, and one of them, having had the same term of study, was examined for a degree, at New York, at the time I was, and rejected. He unfortunately became embarrassed at the commencement of his examination at the gravity and dignity of such an illustrious board of examiners as David Hosack, Wright Post, Samuel Mitchell, Valentine Mott, John W. Francis and others.

I attended two courses of medical lectures, one at New Haven, 1817-18; one at Philadelphia, 1818-19. The professorships at the former school were filled by the venerable Nathan Smith, surgery; Benjamin Silliman, chemistry; Eli Ives, materia medica, and practice; John Knight, anatomy and physiology. At Philadelphia the chairs were filled by Philip Lyng, physiology and surgery; John Lyng Dorsey, anatomy; Nathan Chapman, materia medica; Dr. James, obstetrics and diseases of women; Robert Hare, chemistry; and I think Dr. James Rush was connected in some way with the institution. During the winter Dr. Dorsey died much regretted and highly esteemed by the class which numbered nearly 400.

The old University of Pennsylvania at this period was regarded as the fountain head of American medical literature, though there was a feeling of rivalry between the cities of Philadelphia and New York. The question of a new school, the Jefferson Medical College, began to be agitated in which many of the class participated largely. Of the number was George McClellan, who afterward became a professor in the new school, grew to be a very distinguished surgeon, wrote a book on surgery, but died before he finished it. He left behind a name and fame surpassed by few in the profession. His success was the result of indomitable perseverance and a most happy power to surmount and remove obstacles which would have hopelessly discouraged a more modest and less resolute man. He went to Philadelphia poor, having exhausted his patrimony in his college studies, but found in the city some distant relations of his mother, and threw himself on their patronage, first for a suit of clothes. Soon he succeeded against a half dozen powerful competitors for the place of house physician at the Philadelphia Alms House. This stopped all expense for board, lights, food and servants, living as he did in the family of the steward. As there were nearly 1,000 paupers in the house he had large opportunities to study disease among the living, and an unobstructed chance to practise dissections on the dead. I remember and with pleasure record many acts of personal kindness and courtesy from Mac while we were fellow-students at Philadelphia.

I came to this town, Sardinia, July 3, 1820, at the instance principally of my friends Elihu and Joseph Rice, and Henry Bowen. We had been neighbors in Coventry. Joseph Rice emigrated at the same time and for several days we traveled in company on our journey hither, he with a pair of horses and wagon load of goods and I with a single horse and buggy. The country was very new, the first settlements having been made about 1810, the roads were bad, houses mostly log cabins and the prospect, as I first thought, rather cheerless as a location for a doctor. The news that a new doctor was expected had spread somewhat extensively, and on the 4th, Independence Day, I had a call or two. My calls multiplied rapidly and within a few years my circle embraced a territory about 40 miles in diameter with occasional trips into Northern Pennsylvania, a distance of seventy-five miles. It required almost herculean strength to meet with anything like decent promptness the incessant demands on professional labor, and for much of the time the use of two or three horses. I had the good fortune to buy a black horse brought from Otsego County by Mr. Horace Rider, called "The Captain," a most extraordinary animal. For speed, capacity for endurance, and uninterrupted health, and intelligence I have never seen his equal. He was besides being my family horse, the companion of my professional travels for about twenty years, and for his fidelity as my servant deserves a better monument than this hasty tribute to his memory. He died on the farm at the advanced age of 30 years after serving as my locomotive for a distance, in the aggregate, of some 150,000 miles. Requiescat in Pace "Captain."

If I acquired any noteworthy distinction in my profession it was mainly, I think, as a surgeon. It was my fortune very soon after I commenced practice here, to treat a few very desperate cases of accident. My first was that of Henry Wetherill, a son of Jared Wetherill, who had his skull badly fractured with loss of substance of the brain; one arm fractured above and below the elbow, and one leg, all by the falling of a tree-trunk. He was brought in senseless. After removing a piece of bone, driven deeply into the substance of the brain, and applying the usual dressing to the wound and the broken limbs, he in a few hours became violently crazy which lasted a week, the injury causing violent inflammation of the brain. I bled him freely and repeatedly. In about a week from the date of his injury he became quiet and rational and, contrary to all expectation, recovered rapidly and lead an active and busy life for 25 or more years, dying of edematous inflammation of a leg, I think. The news of Henry's recovery from the broken head was spread by the gossipers over a large territory, often with exaggerations not disadvantageous to me as a surgeon, and was a first stepping-stone to my advancement.

About the same time an accident befell Colonel Josiah Emery, of Aurora. In sliding down from a hay mow, where he had gone to feed his cattle, very early in the morning, he fell astride the handle of his hay fork which stood nearly perpendicular beside the mow. The weight of his body descending with sufficient velocity forced the handle into his body through the perineum nearly or quite a foot, rupturing his bladder, and on being withdrawn leaving inside the bladder a piece of his pantaloons an inch and a half in diameter. I did not reach his house until evening, some 12 hours after the injury. His case had just been abandoned by Dr. Chapin, of Buffalo, as necessarily fatal. The wound of the perineum was now so firmly closed by the increasing swelling of the part as to be impervious to an instrument without great pain, and he could void no urine by the urethra. His abdomen had commenced to swell, and against the remonstrance of Dr. Chapin, I passed a catheter into the bladder by the natural passage with the escape of over a quart of bloody urine. He had instant and great relief. By wearing the instrument a week or two and thus keeping the bladder empty, the two edges healed and in the course of some months, the piece of woolen cloth came away, a thread or two at a time, via urethra, and his recovery was happily most perfect. Colonel Emery is now living after a lapse of almost half a century. I think of those fortunate results with many others I might record, memories of early professional life, with much satisfaction.

I think I must have treated during the almost fifty years that I have practised some twenty-five or thirty cases of fractured skull, many of them as bad as was Wetherill's, all but two or three of which recovered. I must have amputated nearly as many arms and legs with nearly the same success. Many of these patients, to be sure, are now dead but not from any cause connected with the operations.

By an imperfect list which I have preserved for a good while, I must have aided professionally at the birth of over 3.400 children, 38 being twins. Among the number was one case (premature) of four living children at one birth. None of these infants had sufficient vitality to live. (Each parent of this remarkable birth was a twin, a fact of some interest to the naturalist and medical philosopher.) I performed the operation of version of the fetus some dozen times, three times in cases of placenta previa with frightful and exhausting hemorrhage. The mothers all survived, but none of the children. The others were cases of malpresentation, or puerperal convulsions before delivery. The turning and artificial delivery seemed to arrest the convulsions in five or six. Several of the women are now living. With two or three others the results were less fortunate. My rule was never to introduce the hand till the os was soft and dilatable as india rubber.

I have been many times embarrassed when called to cases where the terrible mangling of a limb has forced on me prompt decision of the question of primary amputation. I may have erred. Once I should have erred had not the father of a boy, whose knee joint was torn open and the femur and tibia fractured, refused to have the limb amputated. It was 40 years ago. The boy recovered with a stiff knee and I believe is living now.

Perhaps I should record here my experiences with dislocated joints—hip, shoulder and elbow. The obstacles to reduction most frequently met have been powerful antagonistic muscular contractions. Those I was accustomed to overcome in plethoric, muscular subjects by inducing deliquium by bleeding from a large orifice with the patient in an erect position, preceded by nausea from emetic tartar. By these means I remember to have reduced by my own unaided strength many dislocations that had resisted the combined strength and ingenuity of many physicians for days. Mr. Smith, of Hinsdale, head of humerus in axilla, six men had pulled on it off and on for 24 hours. Deacon Atwater, same place, elbow dislocation. Joseph Oyer, Otto, head of femur in ischiatic notch. Same method.

In my professional and personal intercourse with my medical brethren, I ever aimed to preserve the esprit de corps of the profession. That I sometimes gave offense I fear and now regret it. Some of the warmest and most enduring personal friendships grew up between myself and some of my medical brethren. A nobler man than Dr. Carlos Emmons I never knew. From our respective locations we were necessarily rivals for patronage, yet for forty years not a jealous or unkind thought ever marred our uninterrupted and fraternal exchange of kind offices, and I can never while life lasts cease to remember with the deepest emotion his true, faithful and unfailing support, through evil as well as good report.

I have recently been saved from impending death. My horse took fright and became unmanageable. I was in a carriage and descending a long, steep hill. As soon as the race commenced the velocity became terrific. I was enabled in the emergency to decide on a course, contrary to what I usually regard the safest and always extremely hazardous. I leaped from the flying and doomed vehicle at the only instant of time and at the only point on the way ere it became a complete wreck, at which I could have done so, to all human appearances, without certain destruction. I escaped without serious injury. I have had many such escapes I know, and how many of which I do not know. God only knows. I give very little credit to myself for sagacity or management in such emergencies. In the late one I was as powerless as an infant. I chose to attribute my deliverance to God's providence.

> "Fountain of mercy whose all seeing Eye
> Pervades the soul and reads what passes there;
> Accept my thoughts for thanks, I have no words.
> My soul o'er fraught with gratitude and awe
> Rejects the aid of language.
> Lord, behold my heart."

I have just discharged a patient, Mr. Daniel Andrews, aged 76, with me some weeks to undergo the operation for cataract. He had been blind in one eye twenty years and in the other ten. The operations, two in one eye and one in the other, have been tolerably successful. The old man is almost frantic with joy at again beholding objects and the light of day. On parting he almost choked with emotion and showered his benedictions on me and the family in great profusion. I thought of Him of Nazareth who by the word (?) of his power would not only restore lost vision but say to the dead Lazarus "come forth" and it was so, and I felt humble.

